# Ethno-Specific Risk Factors for Adverse Pregnancy Outcomes: Findings from the Born in Bradford Cohort Study

**DOI:** 10.1007/s10995-016-1936-x

**Published:** 2016-03-16

**Authors:** Tomasina Stacey, Stephanie Prady, Melanie Haith-Cooper, Soo Downe, Nigel Simpson, Kate Pickett

**Affiliations:** School of Healthcare, University of Leeds, Leeds, LS2 9JT UK; Department of Health Sciences, University of York, Heslington, York, Yorkshire YO10 5DD UK; School of Health Studies, University of Bradford, Richmond Road, Bradford, Yorkshire BD7 1DP UK; School of Health, University of Central Lancashire, Brook Building, Preston, PR1 2HE UK; Department of Women’s and Children’s Health, Level 9, University of Leeds, Worsley Building, Clarendon Way, Leeds, LS2 9NL UK

**Keywords:** Small for gestational age, Preterm birth, Born in Bradford, Depression, Financial strain, Ethnic differences

## Abstract

*Objectives* Preterm birth (PTB) and small for gestational age (SGA) are major causes of perinatal mortality and morbidity. Previous studies indicated a range of risk factors associated with these poor outcomes, including maternal psychosocial and economic wellbeing. This paper will explore a range of psycho-social and economic factors in an ethnically diverse population. *Methods* The UK’s Born in Bradford cohort study recruited pregnant women attending a routine antenatal appointment at 26–28 weeks’ gestation at the Bradford Royal Infirmary (2007–2010). This analysis includes 9680 women with singleton live births who completed the baseline questionnaire. Data regarding maternal socio-demographic and mental health were recorded. Outcome data were collected prospectively, and analysed using multivariate regression models. The primary outcomes measured were: PTB (<37 weeks’ gestation) and SGA (<10th customised centile). *Results* After adjustment for socio-demographic and medical factors, financial strain was associated with a 45 % increase in PTB (OR 1.45: 95 % CI 1.06–1.98). Contrary to expectation, maternal distress in Pakistani women was negatively associated with SGA (OR 0.65: CI 0.48–0.88). Obesity in White British women was protective for PTB (OR 0.67: CI 0.45–0.98). Previously recognized risk factors, such as smoking in pregnancy and hypertension, were confirmed. *Conclusions* This study confirms known risk factors for PTB and SGA, along with a new variable of interest, financial strain. It also reveals a difference in the risk factors between ethnicities. In order to develop appropriate targeted preventative strategies to improve perinatal outcome in disadvantaged groups, a greater understanding of ethno-specific risk factors is required.

## Significance

### What is Already Known on this Topic?

A number of risk factors have been identified for small for gestational age and preterm birth. These have included some psycho-social factors. The rates of these adverse outcomes differ between ethnic groups, with the most socially disadvantaged groups being most at risk.

### What this Study Adds

This paper finds an association between maternal financial strain and risk of preterm birth that is as high as that for smoking. It also suggests that there is a difference in risk factors between ethnicities.

Preterm birth (<37 weeks of gestation) (PTB) is a major cause of neonatal and infant mortality and morbidity. In high income countries, there has been minimal decline in the rate of preterm birth in the last few decades [29]. Small for gestational age (SGA) is also associated with an increased risk of perinatal mortality and morbidity, and poor long term health outcomes such as obesity, hypertension and other cardiovascular diseases [4, 9]. The consequences of these poor perinatal outcomes present a significant public health issue, requiring an exploration into primary prevention.

The highest rates of PTB and SGA occur in the most socially disadvantaged groups within the population [30]. In the United Kingdom, South Asian mothers have a slightly higher rate of preterm birth compared to White British mothers and their babies are twice as likely to be born with a low birthweight (below 2500 g) [21]. Known risk factors for SGA and PTB include: smoking, alcohol use, diabetes, hypertension, maternal age and body mass index [13, 15, 27]. Previous studies have suggested that maternal psychosocial health (such as chronic stress and anxiety) may also be associated with PTB and SGA [22]. It is unclear, however, whether these associations are specific to the particular populations studied, or if they are generalizable to other population groups. The development of appropriately targeted prevention strategies requires more analysis of relevant population groups. Our primary aim was therefore to explore ethno-specific risk factors for PTB and SGA, in particular in relation to psychosocial health, within an urban, multi-ethnic, socially disadvantaged cohort.

## Methods

Born in Bradford (BiB) is a longitudinal multi-ethnic community birth cohort study which aims to examine how environmental, psychological and genetic factors impact on maternal and child health and wellbeing [35]. Recruitment took place between 2007 and 2010 at the Bradford Royal Infirmary (BRI). All women who attended a routine glucose tolerance test (offered to all pregnant women at 26–28 weeks’ gestation) were invited to take part in the study and written consent was obtained. Baseline data were collected through an interview administered questionnaire held in a designated room. There were three phases of data collection with slight variants to the baseline questionnaire, with the General Health Questionnaire 28 (GHQ-28) [12] being administered in Phases 2 and 3. The interviews were conducted in English, Mirpuri (a spoken variant of Punjabi) or Urdu.

The questionnaire covered a wide range of socio-economic questions regarding financial security and lifestyle factors. It also included items from the GHQ-28, a commonly used screening tool for psychological distress [12]. Over 80 % of women who attended the clinic were recruited (12,453) and the cohort is broadly representative of the city’s maternal population [35].

Ethics approval for the data collection was granted by Bradford Research Ethics Committee (Ref 07/H1302/112).

### Dependent Variables

PTB was defined as birth occurring at less than 37 weeks’ gestational age, based on the estimated date of delivery calculated by the dating scan (if available), or last menstrual period. SGA was defined as a birthweight less than the 10th customised centile, using GROW software from 2013, https://www.gestation.net/cc/about.htm [8]. These categorisations were derived from maternal characteristics, birthweight and gestational age data recorded in the electronic maternity system (eCLIPSE) at the BRI.

### Independent Variables

Information on diabetes, hypertension, parity and body mass index (BMI) came from eCLIPSE and the remaining data from the baseline questionnaire. Data on diabetes status at booking and any subsequent diagnosis of gestational diabetes were combined to form one binary variable defining diabetic status. Data on hypertension status at booking, subsequent pregnancy-induced hypertension and/or pre-eclampsia were combined in the same way. We calculated BMI using weight and height at first booking and created categories based on the WHO criteria [31]: underweight (<18.5), normal (18.5–24.9), overweight (25–29.9), and obese (≥30). Although there is suggestion that ethnic specific BMI categories might be a more appropriate measure of obesity [32], it has been found in this cohort that lowering the BMI obesity threshold for South Asian women does not improve the predictive ability to identify adverse pregnancy outcomes [6], conventional categories were therefore applied.

The definition of ethnicity in BiB was based on the UK’s 2001 census categories (ONS 2001) and comprised a question asking which ethnic group the mothers considered themselves to belong to. We then classified women as White British, Pakistani or Other ethnic origin (including amongst Other; non-Pakistani Asian, African, other European and Middle Eastern women).

For those women completing the baseline questionnaire in English the GHQ-28 was administered as part of a self-completion module at the end of the interview for participants enrolled during Phases 2 and 3. For those who chose to have the interview in Mirpuri or Urdu, the questions were read aloud and the interviewer coded the response. We did not create a summary score threshold for distress because the measurement properties of the GHQ-28 may not be equivalent between ethnic groups in this cohort [10]. Instead, we scored the instrument using the GHQ methods [25, 26] and derived two indicators of distress. First, we used a non-parametric threshold to indicate women at risk of distress using the first 21 questions (relating to somatic symptoms, anxiety and insomnia, and social dysfunction) and set this at the 85th centile score within each ethno-language group (White British-English, Pakistani-English, Pakistani-Urdu, Pakistani-Mirpuri, other ethnicities-English, all other non-English). Second, we took four out of the seven questions from the Severe Depression subscale of the GHQ-28 which have been found to broadly relate to the same concept across ethno-language groups [25, 26], and created an indicator which we term ‘hopelessness’.

More than 35 % of South Asian women in BiB did not know or did not report their household income, but they were much more likely to answer questions on financial security. “*How well would you say you or you and your husband/partner are managing financially these days?”* We categorised those who reported *‘living comfortably’, ‘doing alright’* or *‘just getting by’*, as financially secure and those who responded ‘*finding it quite difficult’* or ‘*very difficult’* as struggling financially. Financial security has a psychosocial meaning that goes beyond material wealth and involves the extent to which the respondent perceives their income to be enough for the family cost of living.

We created a binary indicator of area deprivation from national quintiles of Index of Multiple Deprivation (IMD), classifying those in the most deprived quintile against all others. In line with other studies, marital status was classified as a binary variable: married and not married (cohabiting and single) [28]. The binary variable for education compared those with fewer than 5 GSCEs, unknown, or equivalent qualification that could not be classified, with those who achieved 5 GCSEs or higher. We also generated binary variables for smoking and drinking alcohol at any time during pregnancy.

### Missing Data

In this analysis we included 9680 singleton non-anomalous births where the mother completed the baseline questionnaire and gave birth at the BRI (78 % of the total cohort). We used the data from the first enrolled pregnancy for women who enrolled in the study more than once during the recruitment period. We did not analyse data from 72 women for whom the language in which the questionnaire was administered was not stated and where we also had no data on ethnicity.

Nearly one-third of women were missing at least one covariate, which we assumed were either randomly missing (e.g. no GHQ-28 in Phase 1) or missing dependent on observed covariates (e.g. women of Pakistani origin less likely to respond about their financial situation). To utilise the entire sample with corrected variance we imputed missing covariate data using chained equations as implemented in Stata 13 (M = 10). We included all covariates and outcomes in our imputation model, along with design variables (questionnaire phase, language of administration). For the ‘risk of distress’ variable, we performed a simple imputation (low score) for those missing up to 4 items (N = 371). We included these along with respondents who had completed all the GHQ-28 data (N = 7765) and categorised risk of psychological distress as a binary variable for all these respondents combined (total N = 8136). We set those missing 4 or more GHQ-28 responses to zero (N = 56), and performed multiple imputation on both these cases and those with all scores missing (N = 1634). Of all the participants in this category, 81.2 % were enrolled in Phase 1 where the GHQ-28 was not presented. For the ‘risk of hopelessness’ variable, we categorised risk on all complete cases (N = 8013, 82.1 %), and imputed risk as a binary variable for all others. For the overall model, we imputed on the whole dataset and included ethnicity as a variable during the imputation process.

### Statistics

We tabulated socio-demographic status by ethnic group. We then fitted unadjusted logistic regression models for the association between a covariate and each outcome of interest (SGA and PTB) for the sample as a whole and stratified by ethnic group. We then fitted fully adjusted multivariate models for the whole sample and also stratified by ethnic group. We calculated odds ratios (OR) with 95 % confidence intervals (CI) and P values, set at <0.05 for statistical significance. In this paper we present models based on the imputed dataset. We ran all models again using data from complete cases (excluding all missing data) finding results broadly similar to the imputed models.

## Results

### Participant Demographics

The demographic characteristics of the cohort are shown in Table 1 with 40·6 % reporting to be White British, 44 % Pakistani and 15.4 % ‘Other ethnicity’. The overall rate of PTB was 5·4 %. A lower proportion of Pakistani women experienced PTB (4·8 %) compared to either White British or Other ethnic groups (5·8 and 5·9 % respectively). The rate of SGA at 16 % was higher than the 10 % baseline and was similarly raised within each ethnic group.

**Table 1:** Demographic data of study population

	White British (N = 3929)		Pakistani (N = 4264)		Other (N = 1487)		Total (N = 9680)	
	N	%	N	%	N	%	N	**%**
SGA < 10th								
No	3274	83.3	3580	84	1274	85.7	8128	84.0
Yes	655	16.7	684	16	213	14.3	1552	16.0
Preterm birth								
No	3702	94.2	4058	95.2	1399	94.1	9159	94.6
Yes	227	5.8	206	4.8	88	5.9	521	5.4
Age								
<20	465	11.8	109	2.6	73	4.9	647	6.7
21–34	2964	75.4	3629	85.1	1219	82	7812	80.7
35+	500	12.7	526	12.3	195	13.1	1221	12.6
Parity								
Nulliparous	1977	52.1	1450	35.6	729	51.1	4156	44.7
1–3	1719	45.3	2303	56.5	659	46.2	4681	50.3
>3	100	2.6	322	7.9	38	2.7	460	4.9
Missing	133	3.4	189	4.4	61	4.1	383	4
BMI								
Underweight	93	2.5	233	5.7	73	5.1	399	4.3
Normal	1658	44.3	1854	45.7	721	50.6	4233	45.9
Overweight	1080	28.9	1207	29.7	375	26.3	2662	28.9
Obese	911	24.3	766	18.9	255	17.9	1932	20.9
Missing	187	4.8	204	4.8	63	4.2	454	4.7
Marital status								
Married	1235	31.5	4151	97.4	1112	74.8	6498	67.2
Not married	2691	68.5	112	2.6	375	25.2	3178	32.8
Missing	3	0.1	1	0	0	0	4	0
Index of Multiple Deprivation								
Quintiles 2–5	1929	49.1	869	20.4	484	32.5	3282	33.9
Quintile 1	1998	50.9	3394	79.6	1003	67.5	6395	66.1
Missing	2	0.1	1	0	0	0	3	0
Education								
Higher education	2764	70.4	2967	69.8	1111	75.1	6842	70.8
Less education	1162	29.6	1285	30.2	369	24.9	2816	29.2
Missing	3	0.1	12	0.3	7	0.5	22	0.2
Migration history								
Born in UK	3808	98.4	1808	43.1	510	35.2	6126	64.4
Migrated before age 16	46	1.2	475	11.3	151	10.4	672	7.1
Migrated ≥ age 16	15	0.4	1911	45.6	788	54.4	2714	28.5
Missing	60	1.5	70	1.6	38	2.6	168	1.7
Smoking in pregnancy								
No	2310	64.4	4048	96.7	1295	90.2	7653	83.1
Yes	1279	35.6	137	3.3	140	9.8	1556	16.9
Missing	340	8.7	79	1.9	52	3.5	471	4.9
Alcohol in pregnancy								
No	1239	31.6	4240	99.7	1112	75	6591	68.3
Yes	2683	68.4	13	0.3	370	25	3066	31.7
Missing	7	0.2	11	0.3	5	0.3	23	0.2
Managing financially								
Yes	3645	93.2	3915	92.4	1329	90.4	8889	92.4
No	268	6.8	324	7.6	141	9.6	733	7.6
Missing	16	0.4	25	0.6	17	1.1	58	0.6
Behind with bills								
No	3371	87.8	3724	91.2	1283	87.9	8378	89.3
Yes	467	12.2	360	8.8	176	12.1	1003	10.7
Missing	91	2.3	180	4.2	28	1.9	299	3.1
Risk of distress								
No	2781	83.0	2889	84.0	1046	84.7	6716	83.7
Yes	569	17.0	551	16.0	189	15.3	1309	16.3
Missing	579	14.7	824	19.3	252	16.9	1655	17.1
Risk of hopelessness								
No	3095	92.4	3002	87.3	1083	87.7	7180	89.5
Yes	255	7.6	438	12.7	152	12.3	845	10.5
Missing	579	14.7	824	19.3	252	16.9	1655	17.1
Diabetes								
No	3719	94.8	3784	88.9	1349	91.0	8852	91.6
Yes	203	5.2	474	11.1	133	9.0	810	8.4
Missing	7	0.2	6	0.1	5	0.3	18	0.2
Hypertension								
No	3483	92.3	3845	94.1	1335	93.7	8663	93.3
Yes	289	7.7	241	5.9	90	6.3	620	6.7
Missing	157	4	178	4.2	62	4.2	397	4.1

More than 20 % of the cohort was classified as obese with the highest rate being amongst White British women (24·3 %). A disproportionate number of women lived in the most deprived quintile as defined by the IMD (66·1 %), including 79·6 % of Pakistani women and 50·9 % of White British women. Almost 17 % of women smoked at some time in the pregnancy, again with a considerable difference seen between White British (35·6 %) and Pakistani women (3·3 %). Diabetes was more prevalent amongst Pakistani compared to White British women, whereas hypertension was slightly less prevalent.

Fewer White British women were at risk of hopelessness compared to either Pakistani or women of other ethnicities. More than 10 % of all participants felt that they were behind with their bills and 7·6 % reported that they were not managing financially.

### Small for Gestational Age

Univariate and multivariate analyses for the whole cohort are presented in Table 2. The evidence found that a number of psychosocial factors were associated with increased risk of SGA on univariate analysis. These associations, however, were not sustained after full adjustment for other variables. In multivariate analysis, hypertension and smoking in pregnancy were both associated with a more than twofold increased risk of SGA and Pakistani ethnicity was found to be associated with a 50 % increased risk of SGA. Diabetes on the other hand was found to be protective of SGA. In addition, in multivariate analysis, women who were at risk of mental distress were found to have a 20 % reduced risk of SGA.

**Table 2:** Logistic regression models for SGA and PTB, whole cohort

	SGA				PTB			
	Unadjusted		Fully adjusted		Unadjusted		Fully adjusted	
	OR	95 % CI	OR	95 % CI	OR	95 % CI	OR	95 % CI
Age								
21–34	0.92	[0.74, 1.14]	1.21	[0.95, 1.53]	0.81	[0.58, 1.12]	1.19	[0.83, 1.71]
35+	0.90	[0.69, 1.16]	1.27	[0.94, 1.70]	0.80	[0.53, 1.19]	1.22	[0.77, 1.94]
Parity								
1–3	1.03	[0.92, 1.15]	1.04	[0.91, 1.18]	**0**.**78**	[**0**.**65**, **0**.**94**]	0.85	[0.69, 1.04]
>3	0.94	[0.72, 1.24]	0.87	[0.64, 1.18]	**0**.**50**	[**0**.**29**, **0**.**84**]	0.48	[0.27, 0.84]
BMI								
Underweight	0.90	[0.67, 1.21]	0.88	[0.66, 1.19]	**1**.**63**	[**1**.**12**, **2**.**39**]	**1**.**68**	[**1**.**14**, **2**.**47**]
Overweight	1.10	[0.96, 1.26]	1.09	[0.95, 1.26]	0.92	[0.73, 1.14]	0.87	[0.69, 1.09]
Obese	**1**.**25**	[**1**.**08**, **1**.**44**]	1.15	[0.99, 1.35]	1.14	[0.90, 1.45]	0.89	[0.69, 1.15]
Ethnicity								
Pakistani	0.96	[0.85, 1.07]	**1**.**51**	[**1**.**23**, **1**.**86**]	0.83	[0.68, 1.00]	0.98	[0.71, 1.36]
Other	**0**.**84**	[**0**.**71**, **0**.**99**]	1.21	[0.97, 1.50]	1.03	[0.80, 1.32]	1.20	[0.86, 1.66]
Migration history								
Migrated before age 16	0.99	[0.80, 1.23]	1.02	[0.81, 1.29]	0.94	[0.66, 1.33]	0.95	[0.65, 1.38]
Migrated ≥ age 16	**0**.**80**	[**0**.**71**, **0**.**91**]	0.85	[0.73, 1.00]	0.82	[0.67, 1.01]	0.79	[0.61, 1.03]
Health behaviours								
Smoking in pregnancy	**2**.**14**	[**1**.**88**, **2**.**44**]	**2**.**40**	[**2**.**04**, **2**.**82**]	**1**.**54**	[**1**.**25**, **1**.**91**]	**1**.**48**	[**1**.**14**, **1**.**92**]
Alcohol in pregnancy	1.06	[0.94, 1.19]	0.98	[0.83, 1.15]	1.08	[0.90, 1.31]	0.94	[0.73, 1.21]
Psycho-social								
Not married	**1**.**29**	[**1**.**15**, **1**.**44**]	1.14	[0.96, 1.35]	**1**.**28**	[**1**.**07**, **1**.**54**]	1.06	[0.81, 1.38]
More deprived IMD	**1**.**23**	[**1**.**10**, **1**.**39**]	1.13	[0.99, 1.28]	**1**.**27**	[**1**.**04**, **1**.**54**]	**1**.**29**	[**1**.**05**, **1**.**59**]
Less education	**1**.**17**	[**1**.**04**, **1**.**32**]	1.06	[0.94, 1.20]	**1**.**23**	[**1**.**02**, **1**.**49**]	**1**.**22**	[**1**.**00**, **1**.**48**]
At risk for distress	0.92	[0.78, 1.08]	**0**.**80**	[**0**.**67**, **0**.**96**]	1.16	[0.90, 1.49]	1.08	[0.82, 1.42]
At risk of hopelessness	**1**.**21**	[**1**.**02**, **1**.**45**]	1.21	[0.99, 1.49]	1.09	[0.80, 1.47]	0.99	[0.72, 1.38]
Not managing financially	**1**.**25**	[**1**.**03**, **1**.**51**]	1.12	[0.91, 1.38]	**1**.**51**	[**1**.**13**, **2**.**01**]	**1**.**45**	[**1**.**06**, **1**.**98**]
Behind with bills	**1**.**31**	[**1**.**10**, **1**.**55**]	1.07	[0.89, 1.29]	1.16	[0.87, 1.53]	1.01	[0.74, 1.37]
Medical conditions								
Diabetes	**0**.**69**	[**0**.**55**, **0**.**85**]	**0**.**68**	[**0**.**54**, **0**.**85**]	**1**.**54**	[**1**.**17**, **2**.**02**]	**1**.**72**	[**1**.**29**, **2**.**30**]
Hypertension	**2**.**17**	[**1**.**80**, **2**.**60**]	**2**.**33**	[**1**.**92**, **2**.**83**]	**3**.**48**	[**2**.**72**, **4**.**46**]	**3**.**67**	[**2**.**83**, **4**.**78**]

The bold represents significant results

### Preterm Birth

There was evidence that diabetes and hypertension in pregnancy were associated with a significantly increased risk of PTB. Also, being underweight and smoking in pregnancy were found to be positively associated with risk of PTB. A number of socio-economic factors showed univariate association with increased risk of PTB. Living in the most deprived quintile and having less education remained significantly associated after adjustment for potential confounders. Furthermore, women who reported that they were not managing financially were found to have a 45 % increased risk of PTB.

## Stratified Analysis

### White British

For White British women, risk of distress was not found to be positively associated with risk of SGA. However, for those who smoked there was an almost threefold increased risk (Table 3). Smoking was also shown to be associated with PTB, although the strength of association was not as strong. In multivariate analysis, obesity in White British women was found to be protective for PTB.

**Table 3:** Risk factors for SGA and PTB, White British women

	SGA				PTB			
	Unadjusted		Fully adjusted		Unadjusted		Fully adjusted	
	OR	95 % CI	OR	95 % CI	OR	95 % CI	OR	95 % CI
BMI								
Underweight	0.73	[0.38, 1.39]	0.60	[0.31, 1.15]	1.83	[0.92, 3.64]	1.72	[0.85, 3.05]
Overweight	1.16	[0.95, 1.43]	1.19	[0.96, 1.48]	0.86	[0.61, 1.20]	0.82	[0.58, 1.16]
Obese	1.14	[0.92, 1.41]	1.11	[0.88, 1.39]	0.84	[0.59, 1.21]	**0**.**67**	[**0**.**45**, **0**.**98**]
Health behaviours								
Smoking in pregnancy	**2**.**81**	[**2**.**35**, **3**.**37**]	**2**.**78**	[**2**.**28**, **3**.**40**]	1.46	[1.11, 1.93]	**1**.**38**	[**1**.**01**, **1**.**88**]
Alcohol in pregnancy	1.06	[0.88, 1.27]	1.03	[0.85, 1.25]	0.91	[0.69, 1.21]	0.94	[0.70, 1.26]
Psycho-social								
Not married	**1**.**60**	[**1**.**32**, **1**.**95**]	1.20	[0.97, 1.50]	1.24	[0.92, 1.67]	1.04	[0.74, 1.46]
More deprived IMD	**1**.**38**	[**1**.**17**, **1**.**64**]	1.10	[0.92, 1.33]	**1**.**43**	[**1**.**09**, **1**.**88**]	**1**.**35**	[**1**.**01**, **1**.**82**]
Less education	**1**.**25**	[**1**.**05**, **1**.**50**]	0.99	[0.81, 1.20]	1.26	[0.95, 1.68]	1.16	[0.86, 1.56]
At risk for distress	1.11	[0.88, 1.40]	0.94	[0.71, 1.23]	1.18	[0.83, 1.69]	1.07	[0.71, 1.63]
At risk of hopelessness	1.34	[0.99, 1.83]	1.17	[0.82, 1.69]	1.23	[0.75, 2.01]	1.09	[0.61, 1.95]
Not managing financially	1.31	[0.96, 1.78]	1.01	[0.72, 1.41]	**1**.**58**	[**1**.**01**, **2**.**48**]	1.54	[0.95, 2.49]
Behind with bills	**1**.**49**	[**1**.**17**, **1**.**89**]	1.12	[0.86, 1.46]	1.12	[0.75, 1.67]	0.93	[0.61, 1.44]
Medical conditions								
Diabetes	0.59	[0.38, 0.93]	**0**.**61**	[**0**.**35**, **0**.**97**]	**1**.**66**	[**1**.**00**, **2**.**74**]	**1**.**85**	[**1**.**10**, **3**.**12**]
Hypertension	**1**.**54**	[**1**.**16**, **2**.**05**]	**1**.**77**	[**1**.**31**, **2**.**39**]	**2**.**41**	[**1**.**63**, **3**.**58**]	**2**.**90**	[**1**.**91**, **4**.**40**]

The bold represents significant results

### Pakistani

In Pakistani women, risk of distress was found to be protective of SGA. This association persisted after full adjustment (Table 4). There was little evidence of an association between other psychosocial factors and SGA. Smoking, diabetes and hypertension were all found to be positively associated with increased risk of preterm birth.

**Table 4:** Risk factors for SGA and PTB, women of Pakistani origin

	SGA				PTB			
	Unadjusted		Fully adjusted		Unadjusted		Fully adjusted	
	OR	95 % CI	OR	95 % CI	OR	95 % CI	OR	95 % CI
BMI								
Underweight	1.06	[0.73, 1.53]	1.07	[0.74, 1.56]	1.35	[0.75, 2.42]	1.34	[0.73, 2.45]
Overweight	1.08	[0.89, 1.32]	1.06	[0.86, 1.30]	0.95	[0.67, 1.35]	0.92	[0.64, 1.32]
Obese	**1**.**25**	[**1**.**00**, **1**.**56**]	1.14	[0.89, 1.45]	1.21	[0.83, 1.76]	0.99	[0.65, 1.50]
Smoking in pregnancy	1.13	[0.72, 1.77]	1.11	[0.70, 1.76]	**2**.**42**	[**1**.**37**, **4**.**27**]	**2**.**26**	[**1**.**24**, **4**.**14**]
Migration history								
Migrated before age 16	1.07	[0.81, 1.40]	1.08	[0.82, 1.43]	0.96	[0.61, 1.50]	1.01	[0.63, 1.61]
Migrated ≥ age 16	0.84	[0.71, 1.00]	0.86	[0.71, 1.03]	0.74	[0.55, 1.00]	0.78	[0.56, 1.07]
Psycho-social								
Not married	1.36	[0.86, 2.17]	1.26	[0.77, 2.06]	1.75	[0.87, 3.52]	1.23	[0.58, 2.61]
More deprived IMD	1.05	[0.85, 1.29]	1.02	[0.83, 1.26]	1.22	[0.84, 1.76]	1.19	[0.82, 1.74]
Less education	1.08	[0.91, 1.29]	1.12	[0.93, 1.35]	1.14	[0.85, 1.54]	1.26	[0.92, 1.73]
At risk for distress	**0**.**71**	[**0**.**54**, **0**.**93**]	**0**.**65**	[**0**.**48**, **0**.**88**]	0.99	[0.64, 1.52]	0.91	[0.58, 1.42]
At risk of hopelessness	1.10	[0.84, 1.45]	1.22	[0.90, 1.65]	1.27	[0.79, 2.06]	1.16	[0.67, 2.01]
Not managing financially	1.16	[0.86, 1.56]	1.23	[0.90, 1.69]	1.31	[0.82, 2.12]	1.24	[0.74, 2.08]
Behind with bills	0.95	[0.71, 1.29]	0.94	[0.68, 1.29]	1.20	[0.75, 1.91]	1.22	[0.74, 2.00]
Medical conditions								
Diabetes	**0**.**74**	[**0**.**56**, **0**.**98**]	**0**.**71**	[**0**.**53**, **0**.**95**]	**1**.**48**	[**1**.**00**, **2**.**19**]	**1**.**59**	[**1**.**04**, **2**.**40**]
Hypertension	**3**.**03**	[**2**.**30**, **4**.**00**]	**3**.**08**	[**2**.**30**, **4**.**10**]	**5**.**27**	[**3**.**65**, **7**.**60**]	**5**.**48**	[**3**.**71**, **8**.**10**]

The bold represents significant results

### Other Ethnic Groups

For women of other ethnic origins, hypertension, smoking in pregnancy and living in the most deprived quintile were all associated with an increased risk of SGA (Table 5). Migrating to the United Kingdom after the age of 16 was found to be protective of SGA. Hypertension in pregnancy and being underweight were both strongly associated with increased risk of PTB for this group, with no other factors reaching statistical significance.

**Table 5:** Risk factors for SGA and PTB, women of other ethnic origin

	SGA				PTB			
	Unadjusted		Fully adjusted		Unadjusted		Fully adjusted	
	OR	95 % CI	OR	95 % CI	OR	95 % CI	OR	95 % CI
BMI								
Underweight	0.67	[0.30, 1.51]	0.67	[0.29, 1.53]	**3**.**33**	[**1**.**55**, **7**.**12**]	**3**.**58**	[**1**.**63**, **7**.**87**]
Overweight	0.92	[0.63, 1.33]	0.90	[0.62, 1.33]	1.01	[0.55, 1.86]	0.93	[0.50, 1.75]
Obese	**1**.**62**	[**1**.**13**, **2**.**34**]	1.45	[0.97, 2.15]	**2**.**33**	[**1**.**33**, **4**.**09**]	1.63	(0.87, 3.05]
Migration history								
Migrated before age 16	0.88	(0.53, 1.46]	0.92	(0.54, 1.56]	0.52	(0.20, 1.35]	0.44	(0.16, 1.21]
Migrated ≥ age 16	**0**.**68**	**(0**.**50**, **0**.**94**]	**0**.**70**	**(0**.**50**, **0**.**98**]	0.99	(0.62, 1.56]	0.94	(0.57, 1.54]
Health behaviours								
Smoking in pregnancy	**2**.**57**	**(1**.**73**, **3**.**82**]	**2**.**62**	**(1**.**62**, **4**.**24**]	0.96	(0.45, 2.04]	0.88	(0.37, 2.10]
Alcohol in pregnancy	0.95	(0.68, 1.33]	0.79	(0.53, 1.18]	1.00	(0.61, 1.65]	1.06	(0.59, 1.90]
Psycho-social								
Not married	1.23	(0.89, 1.70]	0.85	(0.56, 1.28]	1.19	(0.74, 1.92]	1.13	(0.62, 2.04]
More deprived IMD	**1**.**50**	**(1**.**08**, **2**.**09**]	**1**.**45**	**(1**.**02**, **2**.**05**]	1.39	(0.85, 2.26]	1.36	(0.81, 2.28]
Less education	1.22	[0.88, 1.68]	1.10	[0.78, 1.56]	1.44	[0.90, 2.29]	1.47	[0.89, 2.43]
At risk for distress	1.17	[0.78, 1.77]	0.96	[0.60, 1.55]	1.64	[0.96, 2.81]	1.71	[0.92, 3.17]
At risk of hopelessness	1.54	[0.99, 2.38]	1.39	[0.86, 2.27]	1.03	[0.51, 2.11]	0.74	[0.33, 1.66]
Not managing financially	1.43	[0.91, 2.25]	1.13	[0.68, 1.89]	1.72	[0.93, 3.20]	1.54	[0.76, 3.13]
Behind with bills	**1**.**59**	[**1**.**07**, **2**.**38**]	1.19	[0.76, 1.87]	1.18	[0.63, 2.22]	0.92	[0.46, 1.85]
Medical conditions								
Diabetes	0.68	[0.38, 1.21]	0.64	[0.35, 1.17]	**1**.**85**	[**1**.**00**, **3**.**43**]	1.60	[0.81, 3.17]
Hypertension	**2**.**43**	[**1**.**49**, **3**.**96**]	**2**.**32**	[**1**.**38**, **3**.**90**]	**3**.**56**	[**1**.**90**, **6**.**66**]	**3**.**35**	[**1**.**65**, **6**.**80**]

The bold represents significant results

## Discussion

This study provides an insight into different factors (medical, behavioral, and psychosocial) that impact on perinatal outcome in an ethnically diverse and economically deprived population. Overall there was a lower than expected rate of PTB in the cohort (5.6 %) compared to the national average (7.2 %) [24]. This may have been, in part, due to recruitment taking place at around 26–28 weeks’ gestation, excluding women who went into extreme preterm labour and the exclusion of multiple pregnancies from the analysis.

Conversely, there were a greater proportion of SGA babies (16 %) than the national average for the UK population. The higher rate in this sample possibly reflects the high prevalence of risk factors associated with SGA within the pregnant population of Bradford as a whole. Bradford is a socially deprived city, with two-thirds of the population living in the most deprived quintile as defined by the nationally derived IMD.

This study is consistent with other that have described an association between hypertension in pregnancy and both SGA and PTB within all ethnic groups [2]. In line with other studies which have suggested that diabetes in pregnancy increases the risk of indicated preterm birth, we also found an association between diabetes and PTB [37].

Our findings showed a significant association between financial strain (‘*not managing financially’*) and risk of PTB, with a similar degree of association to smoking. Financial concerns have been shown to be independently associated with an increased risk of psychological distress in pregnancy [25, 26]. Although social deprivation has long been associated with poor perinatal outcome [7], no other studies have examined the subjective assessment of financial management and perinatal outcomes. The report by the Royal College of Paediatrics and Child Health highlights the role of poverty and in particular social inequality in increasing the risk of poor perinatal and infant outcomes [34].

The relationship between poor psychological health (depression and stress) and adverse perinatal outcomes, in particular PTB, is becoming an area of increasing interest [16]. Women who show signs of depression as assessed by the Edinburgh Postnatal Depression scale have been found to have an increased risk of PTB and SGA [19]. A recent international cohort study also noted an association between stress and anxiety in pregnancy and an increased risk of SGA [17].

The apparently protective effect of maternal distress on risk of SGA in the Pakistani cohort is surprising, particularly as it was not evident on univariate analysis. An explanation for this may be that the GHQ-28 does not identify maternal distress well at this stage in pregnancy, that there are different cultural norms in the way the distress is described, or that it is a chance finding. The other variable we derived from the GHQ-28 (*risk of hopelessness*) showed trends in the expected direction (distress = adverse birth outcome), and, although we did not use a validated measure of hopelessness and there is likely to be variation in the relationship between hopelessness and mental disorder, there is alignment between the two [5, 36].

A protective association was found between obesity and PTB amongst White British women in this study, but not within the other ethnic groups. Previous studies have shown different findings with regard to the association between obesity and PTB, with some indicating an increased risk [37] and others, a lower risk [18]. It may be that the risks are population specific, relating to different pathophysiological pathways, with a reduced risk for spontaneous PTB and an increased risk for indicated PTB [23]. Therefore, when adjustments are made for related factors such as hypertension and diabetes, as in this study, the protective effect of being obese, but healthy, is more clearly visible than in other studies where reasons for indicated prematurity may not have been excluded from the adjusted analyses.

Smoking is a well-established known risk factor for increased risk of SGA [15]. A high proportion of White British women smoked at some stage in their pregnancy (35 %), and this was found to be the most significant risk factor for SGA amongst this population RR 2.78 (CI 2.28–3.40). However in the Pakistani population (where only 3.3 % of women smoked), no association was found RR 1.11 (0.70–1.76).

Migration after the age of 16 was also found to be protective of SGA for non-Pakistani ethnic groups. A ‘healthy migrant effect’, which suggests that babies born to foreign-born women may have better outcomes than those born to ‘native-born’ women, has been previously described and debated [3, 33] and may relate to differences in health behaviour amongst newer migrants [14]. The findings from this study reinforce the need for careful analysis of the ethnic, geographic and socio-economic context of the populations observed.

## Strengths and Limitations

This was a large multi-ethnic cohort study which was shown to be representative of the population of Bradford as a whole. Data were collected prospectively and linked datasets allowed for the collection of relevant perinatal outcomes. This allowed us to control for a wide range of covariates. Although a number of perinatal outcomes were captured, it was not possible to distinguish between spontaneous and medically indicated preterm birth which limited detailed analysis of specific risk factors. In addition, there were a number of missing data, particularly relating to the GHQ-28, and the process of imputation may have impacted on the findings; although when we ran models using only those with the complete dataset, the findings were not significantly altered. A further limitation to this study is that responses to the GHQ-28 in multi-ethnic populations may vary between different ethnic groups and language of administration, independent of the level of actual distress [25, 26]. Other research has reported variation in the expected psychometric properties of the GHQ-28 in pregnant Nigerian women [1] and reduced reliability of the questionnaire when applied late in pregnancy [20]. We potentially mitigated language and interpretation effects by deriving centiles from scores computed within ethno-language groups to categorise risk of distress, and limited the risk of hopelessness variable to questions that had been shown to relate to similar concepts across ethno-language groups [25, 26]. We cannot, however, rule out the possibility that increased measurement error due to pregnancy, or our multi-ethnic sample, or both factors, affected our results.

## Conclusion

This study confirms certain known risk factors for adverse pregnancy outcome. However, it also identifies some previously undocumented and unexpected findings. This includes an association between maternal financial strain and risk of PTB that is as high as that for smoking, which, if generalisable, has important social implications. It also discerns additional relationships in specific subgroups: an unexpectedly lower rate of SGA in the offspring of women of Pakistani origin who reported distress and a reduced rate of PTB amongst White British women who were obese. There is a need for further ethnic-specific studies to understand the mechanistic pathways for psychosocial stress and poor pregnancy outcome in order to better inform public health policy.
